# A Systematic Review and Meta-Analysis on the Role of Bempedoic Acid in Cardiovascular Outcomes for Patients With Statin Intolerance

**DOI:** 10.7759/cureus.61572

**Published:** 2024-06-03

**Authors:** Mohamed H Serour, Musab Egaimi, Zahid Khan

**Affiliations:** 1 Cardiology, Sheikh Khalifa Specialty Hospital, Ras al Khaimah, ARE; 2 Medicine, Royal College of Physicians of Edinburgh, Edinburgh, GBR; 3 Acute Medicine, Mid and South Essex NHS Foundation Trust, Southend on Sea, GBR; 4 Cardiology, Barts Heart Centre, London, GBR; 5 Cardiology and General Medicine, Barking, Havering and Redbridge University Hospitals NHS Trust, London, GBR; 6 Cardiology, Royal Free Hospital, London, GBR

**Keywords:** high-statin therapy, statin use, statin intolerance, adverse cardiovascular outcomes, cardiovascular disease risk factor, " "atherosclerotic cardiovascular disease, cardiovascular disease prevention, pattern of dyslipidemia, hyperuricemia. dyslipidemia. bmi .fbs . cardiovascular risk, bempedoic acid

## Abstract

Atherosclerosis, a multifaceted pathogenic process affecting the arteries and aorta, poses a significant threat because of its potential to impede or entirely obstruct blood flow by narrowing blood vessels. This intricate progression involves various factors such as dyslipidemia, immunological responses, inflammation, and endothelial dysfunction. The initial phase manifests as the formation of fatty streaks, considered a pivotal hallmark in the inception of atherosclerotic plaques, a process that can commence as early as childhood. Over time, this process evolves, characterized by the thickening of the arterial inner layer (intima) and accumulation of lipid-laden macrophages, commonly known as foam cells, along with the buildup of the extracellular matrix. Subsequent stages witness the proliferation and aggregation of smooth muscle cells, culminating in the formation of atheroma plaques. As these lesions progress, apoptosis can occur in the deeper layers, further recruiting macrophages, which may undergo calcification and transform into atherosclerotic plaques. Notably, mechanisms such as arterial remodeling and intraplaque hemorrhage also contribute significantly to the progression of atherosclerotic cardiovascular disease, although these facets fall beyond the scope of this article. This study aimed to systematically review and conduct a meta-analysis of randomized controlled trials investigating the efficacy and safety of bempedoic acid in statin-intolerant patients with hyperlipidemia and to provide conclusions and recommendations accordingly. A systematic search of databases, such as PubMed, Web of Science, and Embase, will be performed. Only randomized trials will be included comparing bempedoic acid with placebo in statin-intolerant patients. This study aimed to provide a comprehensive understanding of the role of bempedoic acid in managing hyperlipidemia in statin-intolerant patients. In primary prevention, for patients unable to tolerate recommended statins, bempedoic acid was associated with a significant reduction in major adverse cardiovascular events (MACE) as the primary endpoint.

## Introduction and background

Cardiovascular diseases (CVDs) are the foremost threat to global health, claiming an alarming 17.9 million lives annually. The term CVD encompasses a spectrum of disorders affecting the heart and blood vessels, ranging from coronary heart disease to stroke and rheumatic heart conditions. Shockingly, more than four out of five deaths due to CVD stem from heart attacks and strokes, and what is even more concerning is that one-third of these fatalities occur prematurely in individuals under 70 [1]. This grave reality underscores the urgent need for widespread awareness, preventative measures, and enhanced medical interventions to mitigate the risk factors and offer timely care. Addressing lifestyle choices, managing risk factors, and advocating accessible healthcare are crucial to combating this pervasive global health crisis. Preventing premature deaths due to CVD relies significantly on the identification of individuals at the highest risk and ensuring that they promptly receive suitable treatment. This proactive approach saves lives and fosters a culture of comprehensive healthcare that prioritizes prevention and effective treatment, thereby significantly reducing the burden of CVDs within communities [2].

Atherosclerosis, a multifaceted pathogenic process affecting arteries and the aorta, poses a significant threat due to its potential to impede or entirely obstruct blood flow by narrowing blood vessels. This intricate progression involves various factors such as dyslipidemia, immunologic responses, inflammation, and endothelial dysfunction. The initial phase manifests as the formation of fatty streaks, considered a pivotal hallmark in the inception of atherosclerotic plaque, a process that can commence as early as childhood. Understanding and addressing CVD require comprehensive evaluation and treatment approaches [1].

Prevalence of dyslipidemia

CVD is a leading cause of premature mortality and disability in Europe, and its incidence is increasing in developing countries. The economic burden of CVD in the European Union is substantial, with annual costs exceeding 192 billion euros. The primary clinical manifestations of CVD include coronary artery disease, ischaemic stroke, and peripheral arterial disease. These conditions have complex, multifactorial causes, with lifestyle factors such as smoking, physical inactivity, and dietary habits contributing to the risk. Other risk factors, such as high blood pressure, type 2 diabetes, and abnormal lipid profiles (dyslipidemia), can be modified through medical intervention, while factors such as age and sex are non-modifiable; these guidelines focus on the crucial management of dyslipidemia as a central component of CVD prevention [3].

LDL-C levels tend to follow a normal distribution in the population, slightly skewed to the right. The mean LDL-C levels are approximately 2.6, 3.2, and 3.5 mmol/L (100, 124, and 135 mg/dL) for European women and 2.9, 3.4, and 3.3 mmol/L (112, 132, and 138 mg/dL) for men in the 20-39, 40-65, and 66-100 years age groups, respectively. The 95th percentile value roughly corresponds to an LDL-C level of 5.0 mmol/L (194 mg/dL) [4].

Effects of hyperlipidemia on myocardial function, cardiac electrophysiology, and heart failure

In individuals with familial hypercholesterolemia (FH), various measures of cardiac function, such as endocardial longitudinal strain (LS), myocardial LS, average LV LS, and circumferential strain (CS), significantly decreased. These reductions were correlated with elevated low-density lipoprotein cholesterol-C (LDL-C) levels. Moreover, disruptions in heart function are evident at an early age in FH patients with high total cholesterol (TC) levels. Additionally, diabetic patients with hyperlipidemia were found to have a higher prevalence of left ventricular (LV) hypertrophy than those with diabetes alone. Animal models of hyperlipidemia demonstrated cardiac fibrosis and LV diastolic dysfunction induced by a high-fat and high-cholesterol (HFHC) diet in SHRSP5/Dmcr rats. In mice subjected to an eight-week high-fat and high-sugar diet, a significant reduction in the LV ejection fraction (EF) was observed, accompanied by increases in isovolumic relaxation time, myocardial performance index, and LV end-diastolic pressure. This indicates damage to both cardiac systolic and diastolic functions. Interestingly, transitioning to a standard diet partially reversed contraction and diastolic dysfunction of the heart. Additionally, in hyperlipidemic rabbits, a two-week treatment with high-density lipoprotein (HDL) mimetic peptides significantly improved LV diastolic function [4].

P-oxy phosphatase 1 (PON1) plays a role in inhibiting the oxidation of lipids such as LDL. Interestingly, patients with heart failure exhibited higher levels of oxidized LDL (Ox LDL) in the left ventricle (LV), and this increase correlated with decreased EF and PON1 activity in the LV. Chronic inflammation, which contributes to increased oxidative stress and injury, is associated with the progression of heart failure. As an oxidative product of TC, 7-ketone cholesterol (7KCh) induces oxidative stress in cardiomyocytes. Recent findings have demonstrated higher levels of oxidized 7KCh in red blood cells of heart failure patients than in plasma. The accumulation of 7KCh in cells results in reactive oxygen species formation and cardiomyocyte death, potentially mediated by the Activating Transcription Factor 4 (ATP4)/CHOP pathway. These results suggest that red blood cells may transport 7KCh to the heart tissues, causing direct damage to cardiac cells. However, the causal relationship between increased erythrocyte 7KCh levels and the development of heart failure remains unclear [4].

Lipid metabolism

The two most clinically significant plasma lipids are cholesterol and triglycerides (TGs). Cholesterol plays an essential role in cellular membranes; serves as a precursor for synthesizing steroid hormones, bile acids, and oxysterols; and modifies signaling molecules in neurons. TGs serve as energy sources for the muscle and adipose tissue. These lipids circulate within the hydrophobic core of spherical lipoprotein particles encapsulated by surface phospholipids and apolipoproteins, such as chylomicrons (CM), very LDL (VLDL), intermediate-density lipoprotein (IDL), LDL, and HDL. These distinct lipoproteins are characterized by their size, density, relative lipid content, and specific apolipoproteins, which provide structural stability and function as ligands for receptors and cofactors for processing and transporter molecules [5].

Most of the plasma cholesterol originates from hepatic synthesis, with only 15% to 20% originating from dietary sources. Dietary cholesterol is absorbed by enterocytes in the upper small intestine via the Niemann-Pick C1-like 1 transporter. Within the liver, cholesterol can be obtained from plasma through lipoprotein uptake or synthesized de novo via a multistep process, in which the enzyme 3-hydroxy-3-methylglutaryl CoA reductase (HMGCR) plays a pivotal role. Hepatic free cholesterol is converted to cholesterol ester (CE) for transportation within lipoproteins [5].

Hyperlipidemia is a significant risk factor for CVD. Statins are the first-line therapy for lowering LDL-C levels [6]. However, a subset of patients cannot tolerate statins because of their side effects, necessitating alternative treatments. The prevalence of statin intolerance varies widely in the literature, ranging from 5% to 20%, depending on the definition used [7]. Statin intolerance is generally defined as the inability to tolerate at least two different statins at the lowest daily starting dose owing to the occurrence of symptoms or laboratory abnormalities attributable to the initiated statin therapy. The symptoms may be musculoskeletal (e.g., myalgia and muscle weakness), gastrointestinal, or involve the central nervous system (e.g., sleep disturbances and cognitive effects) [8]. There are two main classifications of statin intolerance: muscle-related statin intolerance (MRSI) and non-muscle-related statin intolerance.

Statin intolerance poses a significant challenge in the management of patients at a high risk of cardiovascular events, as it often leads to discontinuation of therapy, suboptimal treatment, and consequently, inadequate control of LDL-C levels [8]. Therefore, alternative treatments are needed for this patient population. Bempedoic acid, an oral LDL-C-lowering agent has shown promise in this patient population. This study aimed to systematically review the efficacy and safety of bempedoic acid in statin-intolerant patients.

## Review

Statin intolerance is a notable cause of non-adherence to dyslipidemia management. Statin intolerance refers to adverse events (AEs) or laboratory abnormalities attributed to statin treatment that lead to discontinuation. Muscle-related symptoms, notably pain, are the primary cause of discontinuation, with a significant proportion of patients experiencing muscle pain [9].

The importance of distinguishing true statin intolerance from symptoms unrelated to statin use, including the consideration of the *nocebo effect*, where symptoms arise from patient expectations is emphasized. Various studies, including the GAUSS-3 trial, explored statin intolerance and challenged the assumption that muscle complaints are always associated with statin use [10]. The criteria for defining statin intolerance, such as symptoms occurring after initiating therapy, improvement with statin discontinuation, and reappearance upon statin reintroduction, have been established [9]. The classification of statin-associated muscle symptoms (SAMS) into myalgia, myopathy, myositis, and myonecrosis, providing a comprehensive understanding of these conditions, is also discussed [11].

Effective management of statin intolerance is crucial to prevent premature discontinuation of statin treatment, particularly in high-risk patients with muscle complaints. Educating patients about the proven cardiovascular benefits of statins is paramount, emphasizing that myopathy, a rare AE, should not overshadow these advantages [12].

SAMS present a complex interplay of factors influencing their occurrence, demanding thorough consideration in clinical evaluation. Among the myriad risk factors identified, sex plays a role, with women potentially exhibiting increased susceptibility to SAMS [13]. Advanced age, which is not inherently a deterrent to statin tolerance, may become a contributing factor to age-related physiological changes. Conditions such as abdominal obesity and metabolic syndrome, along with frailty, amplify this risk, emphasizing the need for a nuanced approach in these populations [13].

ETC-1002 (bempedoic acid) represents a significant advancement in the field of lipid-lowering agents and offers a safe and effective solution for managing hypercholesterolemia. This medication has demonstrated its potential to substantially reduce key lipid parameters, including TC, LDL-C, non-HDL-C, apolipoprotein B (apoB), and high-sensitivity C-reactive protein (hs-CRP). Notably, this study addressed two critical patient populations. First, for individuals who experience statin intolerance, bempedoic acid provides a much-needed alternative, allowing them to manage their cholesterol levels without adverse effects associated with statins. Second, for patients at high cardiovascular risk who struggle to achieve their target LDL-C levels despite maximally tolerated lipid-lowering therapy, which includes statins and ezetimibe, bempedoic acid serves as a valuable addition to their treatment regimen [14,15].

Bempedoic acid is an oral, once-daily, first-in-class adenosine triphosphate (ATP)-citrate lyase (ACL) inhibitor that reduces cholesterol biosynthesis and lowers LDL-C by upregulating the LDL receptor. It is primarily metabolized in the liver, and its activation is dependent on very long-chain acyl-CoA synthetase-1 (ACSVL1), an enzyme that is not present in skeletal muscles, which may explain the lower incidence of muscle-related side effects [15]. Bempedoic acid has been shown to lower LDL-C levels by 17% to 18% when used as monotherapy and by 28% when combined with ezetimibe in patients with statin intolerance [16].

The CLEAR outcomes trial aimed to determine whether bempedoic acid reduces the incidence of adverse cardiovascular events in patients with high vascular risk, documented statin intolerance, and elevated LDL-C levels. Bempedoic acid has been shown to lower LDL-C levels by 17% to 18% when used as monotherapy and by 28% when combined with ezetimibe in patients with statin intolerance [17]. In addition, a study on patients with atherosclerotic cardiovascular disease (ASCVD) not at the LDL-C goal with maximally tolerated statins suggested that the addition of a bempedoic acid plus ezetimibe fixed-dose combination could provide incremental absolute reductions in MACEs [18,19].

A systematic review by Lin et al. raised concerns regarding the potential unfavorable effects of bempedoic acid on muscular disorders, renal function, and gout [20]. However, this review had a relatively short follow-up period and the long-term safety of bempedoic acid remains uncertain. Furthermore, a review by Nissen et al. showed a reduction in MACEs with bempedoic acid, but the study was limited by a lack of data on patient-reported outcomes and quality of life [10,21].

The long-term safety and efficacy of bempedoic acid have also been evaluated, with a study showing that it was generally well-tolerated and demonstrated sustained efficacy for up to 2.5 years of continuous treatment [16].

ESP55016 (bempedoic acid) generally demonstrates minimal adverse effects, with notable concerns including hyperuricemia, gout, and tendon rupture. Elevated serum uric acid levels have been observed and attributed to competition for the renal OAT2 transporter between uric acid and the glucuronide metabolite of bempedoic acid. Muscle-related adverse effects were not statistically significant when compared with placebo or when co-administered with maximally tolerated statins. The liver-specific metabolism of bempedoic acid contributed to the limited occurrence of muscle-related adverse effects. Rare adverse effects included benign prostatic hyperplasia (1.3% incidence in men when co-administered with high-dose statins) and atrial fibrillation (1.7% incidence when co-administered with high-dose statins). Additionally, there have been instances of increased blood urea nitrogen (4%), serum creatinine (2%), and hematologic effects, such as anemia, leukopenia, and thrombocythemia, although further research is needed to confirm their prevalence and significance [15].

However, the cost-effectiveness of bempedoic acid varies substantially with concurrent statin use and only meets conventional cost-effectiveness thresholds among patients unable to take statins owing to severe side effects [16-22].

In summary, bempedoic acid, an oral cholesterol synthesis inhibitor, is considered a pioneering medication for treating hypercholesterolaemia. Functioning as a pro-drug necessitates activation by the hepatic enzyme 'very long-chain acyl-CoA synthetase-1'. This selective activation within hepatocytes aims to mitigate the risk of muscle-related AEs, a known issue associated with statins. As an ACL inhibitor, bempedoic acid blocks cholesterol synthesis before HMG-CoA reductase, the target of statins. This inhibition leads to reduced cholesterol production and increased LDL receptor expression in hepatocytes, thereby decreasing circulating LDL-C levels. Additionally, bempedoic acid boosts the activity of 5ʹ-adenosine monophosphate-activated protein kinase (AMPK), which curtails the synthesis of glucose and lipids, aiding the reduction of LDL-C through an alternative pathway [23].

Rationale for the systematic review

The recent publication of the randomized controlled trial (RCT) "Bempedoic Acid and Cardiovascular Outcomes in Statin-Intolerant Patients" provided significant new data on the efficacy of bempedoic acid in reducing MACEs among statin-intolerant patients [10,21]. This pivotal study, with its robust design and large sample size, has the potential to significantly influence clinical practice and guidelines. However, while RCT provides valuable insights, it is essential to integrate these findings within the context of a broader body of evidence. Systematic reviews and meta-analyses conducted before this RCT have shown mixed results, with some raising concerns about the potential unfavorable effects of bempedoic acid on muscular disorders, renal function, and gout [20]. Furthermore, these reviews did not have the benefit of including the data from the recent RCT by Nissen et al. trial. To the best of our knowledge, no meta-analysis as of July 2023 has included this RCT [19,20].

Therefore, there is a need for a new literature review that includes the results from this study and previously published RCT to analyze the available evidence, as well as other studies published since the last review. This provides a more comprehensive and updated understanding of the safety and efficacy of bempedoic acid in statin-intolerant patients.

Methodology

Data Collection

In this systematic review and meta-analysis, a thorough search was carried out in line with the Preferred Reporting Items for Systematic Reviews and Meta-Analyses (PRISMA) guidelines across various electronic databases and online repositories, including PubMed, Google Scholar, Web of Science, Embase, and Cochrane Library up to August 2023. Additionally, a manual exploration of other relevant sources was conducted to identify applicable trial studies. The search was confined to studies published in the English language.

Search Strategy

The search strategy was meticulously designed to identify studies relevant to the efficacy and safety of bempedoic acid in statin-intolerant patients with hyperlipidemia. The following MeSH terms and keywords were used in varying combinations across the databases.

Drug-related terms: Bempedoic acid, ETC-1002, ESP55016, ESP-55 016. These terms were chosen to encompass the primary drug of interest in terms of its various nomenclatures and developmental identifiers.

Condition-related terms: Hypercholesterolemia, hyperlipidemia, dyslipidemia, and LDL-C. These terms were selected to ensure the inclusion of studies that focused on the spectrum of lipid disorders, particularly those associated with elevated LDL-C levels. Additional Specific Criteria: Statin intolerance. This term was included to specifically identify studies that examined alternatives to statins, focusing on the role of bempedoic acid in this context.

The search strategy involved using Boolean operators to broaden or refine the search as necessary. For instance, in PubMed, the search was structured as follows. (Bempedoic acid OR ETC-1002 OR ESP55016 OR ESP-55 016) AND (hypercholesterolemia OR hyperlipidemia OR hyperlipidemia OR dyslipidemia OR dyslipidemia OR low-density lipoprotein cholesterol) AND statin intolerance.

Eligibility Criteria

The inclusion and exclusion criteria were determined using the Population, Intervention, Comparison, and Outcomes (PICO) tool, an appropriate tool for this type of research seeking to synthesize evidence from quantitative data (Tables 1-2).

**Table 1:** Inclusion criteria. PICO, Population, Intervention, Comparison, and Outcomes; MACE, major adverse cardiovascular event

Inclusion PICO criteria	Description
Participants	Adult (≥18 years) statin-intolerant patients
Intervention	Receiving bempedoic acid
Comparators	Controls were not receiving bempedoic acid. This study also included patients who received the usual standard of care
Outcome	Studies that included cardiovascular outcomes, such as three-point MACE consisting of cardiovascular death, non-fatal myocardial infarction (MI), or nonfatal stroke
Study design	Randomized controlled trials (RCTs) published in English only

**Table 2:** Exclusion criteria PICO, Population, Intervention, Comparison, and Outcomes

Exclusion PICO criteria	Description
Participants	Adult (≥18 years) patients who were not statin-intolerant
Intervention	Not receiving bempedoic acid
Comparators	Not available
Outcome	Studies that did not include cardiovascular outcomes as endpoints
Study design	All nonrandomized controlled studies, case reports, and editorials will be excluded if they do not specifically include statin-intolerant patients and studies are not in English.

Endpoints

The primary endpoint was four-point MACEs consisting of cardiovascular death, non-fatal myocardial infarction (MI), or non-fatal stroke. Secondary endpoints included changes in LDL-C levels, other lipid parameters, such as triglycerides (TGs) and HDL-C, and AEs.

Data Management

All studies that fulfilled the eligibility criteria were initially manually scrutinized by examining the titles and abstracts to eliminate any obvious duplicates and irrelevant studies. Following this, the articles were amalgamated using reference management software to ensure that there are no repeated studies. For a more detailed evaluation of the eligibility criteria, the full texts of studies that potentially met the criteria were thoroughly examined to discard any irrelevant ones. Any disagreements regarding the data were resolved through consensus after discussion with the tutor.

The extracted data included the following summary data: sample characteristics (specifically focusing on statin-intolerant patients), sample size, interventions (specifically focusing on bempedoic acid treatment), comparators, outcomes (specifically focusing on cardiovascular outcomes such as three-point MACE consisting of cardiovascular death, nonfatal MI, or nonfatal stroke), and timescales to reflect the duration of the study. This approach ensures that the collected data are relevant and specific to the research question at hand.

Statistical Analysis

Statistical analysis involved conducting a meta-analysis using random-effects models to account for potential heterogeneity across studies. The degree of heterogeneity was quantified using the *I*^2 ^statistics, which provided an estimate of the percentage of total variation across studies owing to heterogeneity rather than chance.

Risk of Bias in Individual Studies

The risk of bias for each included study was independently assessed using the Cochrane Collaboration tool for assessing the risk of bias. This tool provided a comprehensive framework for assessing potential sources of bias in clinical trials, including selection, performance, detection, attrition, reporting, and other biases [24].

Results

**Figure 1:**
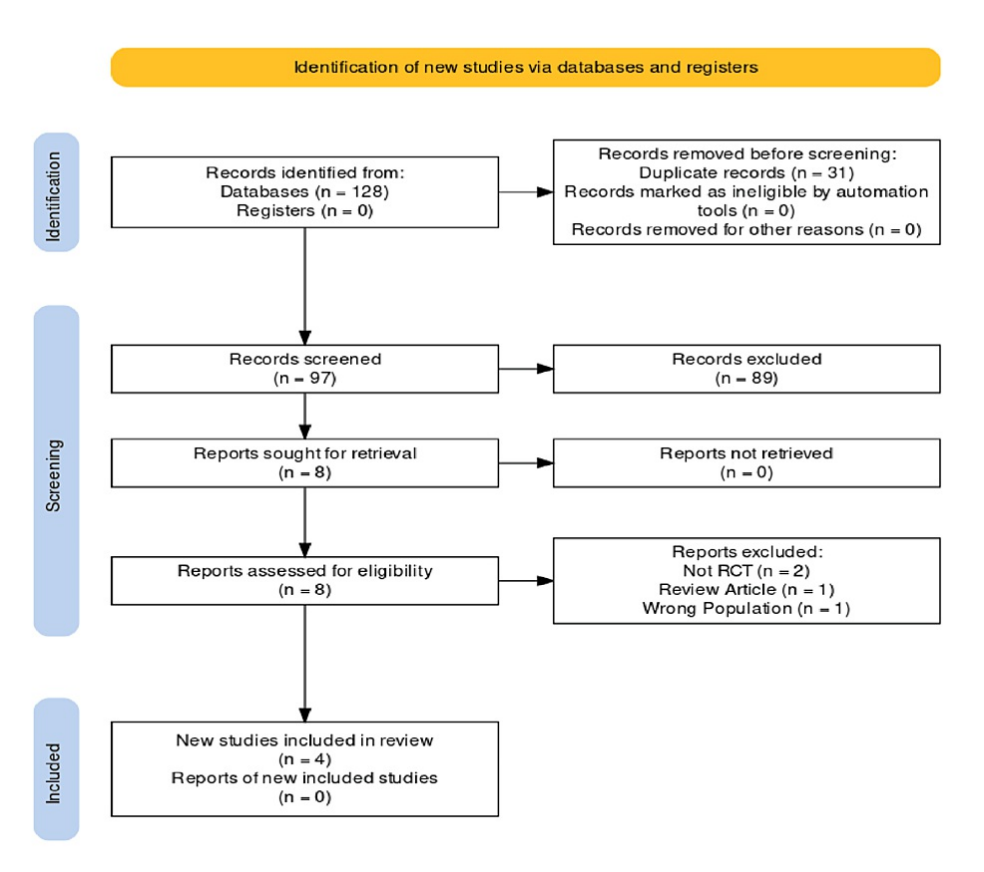
Preferred Reporting Items for Systematic Reviews and Meta-Analyses (PRISMA) flowchart.

A systematic search across databases and registers, as illustrated in the PRISMA flow diagram (Figure 1), yielded a total of five records. Before the screening, 31 duplicate records were removed. The screening process involved 97 records, 89 of which were excluded as duplicates. Following the initial screening, eight reports were identified for full-text retrieval, all of which were successfully obtained and assessed for eligibility. Of these, four reports were excluded for the following reasons: two were not RCTs, one was a review article, and one studied the wrong population, leaving four studies that met the inclusion criteria for this systematic review.

Baseline Characteristics

Four studies were included this in this review. These studies were published between 2018 and 2023 and included studies by Ballantyne et al., Laufs et al., Nissen et al., and Nissen et al. [10,17,21,25].

The first study included 181 participants in the BA arm with an average age of 63.8 years, 60.2% of whom were female, and 27.1% had ASCVD. The placebo arm included 88 participants with an average age of 63.7 years, 63.6% female, and 25.0% with ASCVD [17].

The second study included 234 participants in the BA arm, with an average age of 65.2 years and 56.8% female participants. The placebo arm had 111 participants, averaging 65.1 years of age with 55.0% female representation. Both arms had a 27.1% prevalence of ASCVD [25].

The third study assessed the general efficacy of bempedoic acid across all trial participants, including both primary and secondary prevention patients, resulting in a reduction of LDL-C by about 29.2 mg/dL. Additionally, it reduced MACE (11.7% in the bempedoic acid group vs. 13.3% in the placebo group). The bempedoic acid group included a substantial 6,992 participants with a mean age of 65.5±9.0 years, of whom 48.1% were female. ASCVD was present in 70.0% of participants [10].

The fourth study evaluated the primary prevention efficacy of bempedoic acid specifically in patients with no previous cardiac event, focusing solely on primary prevention patients. It resulted in a reduction of LDL-C by about 30.2 mg/dL. Furthermore, there was a significant reduction in MACE, with rates of 5.3% in the bempedoic acid group compared to 7.6% in the placebo group [21].

The prevalence of diabetes mellitus (DM) ranged from 19.3% in the Ballantyne et al. study to 45.0% in the Nissen et al. study [10,17]. Hypertension (HTN) was also reported, with a prevalence of over 61.3% in the BA arm [17]. Body mass index (BMI) was consistent with an overweight cohort across studies, with BMI values such as 29.5 kg/m² in Ballantyne et al. and 29.9 ± 5.2 kg/m² in Nissen et al. [10,17].

Chronic kidney disease (CKD) prevalence varied and was reported as 75.2% in one study [17]. Baseline lipid profiles, including TC, LDL-C, HDL-C, non-HDL-C, and TGs, exhibited diversity among the participant groups. For instance, LDL-C levels spanned from 123 mg/dL in the placebo arm [10] to 158.5 mg/dL in another study [25].

The primary outcome of two studies was the change in LDL-C levels [17,25], whereas the other two studies focused on a four-component MACE outcome, as shown in Table 3 [10,21].

**Table 3:** Baseline characteristics of included studies. BA, bempedoic acid; N, number; HTN, hypertension; DM, diabetes mellitus; TC, total cholesterol; BMI, body mass index; ASCVD, atherosclerotic cardiovascular disease; CKD, chronic kidney disease; LDL-C, low-density lipoprotein cholesterol; HDL-C, high-density lipoprotein cholesterol; TG, triglycerides; MACE, major adverse cardiovascular event

Study	Nissen et al. [10]		Ballantyne et al. [17]		Nissen et al. [21]		Laufs et al. [25]	
Year	2023		2018		2023		2019	
Arms	BA	Placebo	BA	Placebo	BA	Placebo	BA	Placebo
Total number	6992	6978	181	88	2100	2106	234	111
Age (years)	65.5 ± 9.0	65.5 ± 8.9	63.8	63.7	67.9 ± 6.9	68.5 ± 6.8	65.2	65.1
Female (%)	48.1	48.4	60.2	63.6	58.8	59.2	56.8	55
ASCVD (%)	70	69.8	27.1	25	0	0	27.1	25.3
DM (%)	45	46.3	19.3	19.3	65.2	67	26.9	23.4
HTN (%)	-	-	61.3	58	88.2	88	67.5	67.6
BMI (kg/m^2^)	29.9 ± 5.2	30.0 ± 5.2	29.5	30.5	30.2 ± 5.3	30.4 ± 5.4	30.1	30.6
CKD (%)	20.6	20.7	75.2	80.7	7	7.4	75.2	85.6
TC (mg/dL)	223.5 ± 40.6	223.3 ± 41.1	218.2	208.6	228.5 ± 40.2	229.1 ± 42.3	245.7	241.1
LDL-C (mg/dL)	139.0 ± 34.9	139.0 ± 35.2	129.8	123	142.2 ± 34.5	142.7 ± 35.9	158.5	155.6
HDL-C (mg/dL)	49.6 ± 13.3	49.4 ± 13.3	55.8	57.1	51.1 (13.5)	50.9 (13.7)	52.2	50.4
Non-HDL-C (mg/dL)	173.8 ± 39.5	173.9 ± 40.2	162.4	151.6	177.4 ± 38.7	178.2 ± 41.2	193.5	190.7
TG (mg/dL)	159.5	158.5	135.5	153	162	161.5	156.5	164
Primary outcome	LDL-C change	Four-component MACE	Four-component MACE	LDL-C change	Four-component MACE	Four-component MACE	Four-component MACE	Four-component MACE

Risk-of-Bias Analysis

**Figure 2:**
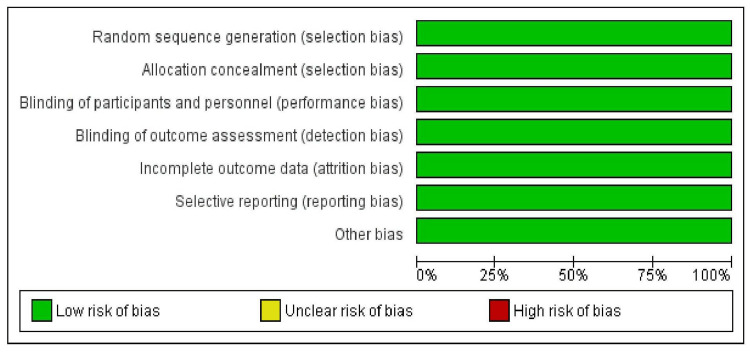
Risk-of-bias summary plot for studies included in the meta-analysis.

**Figure 3:**
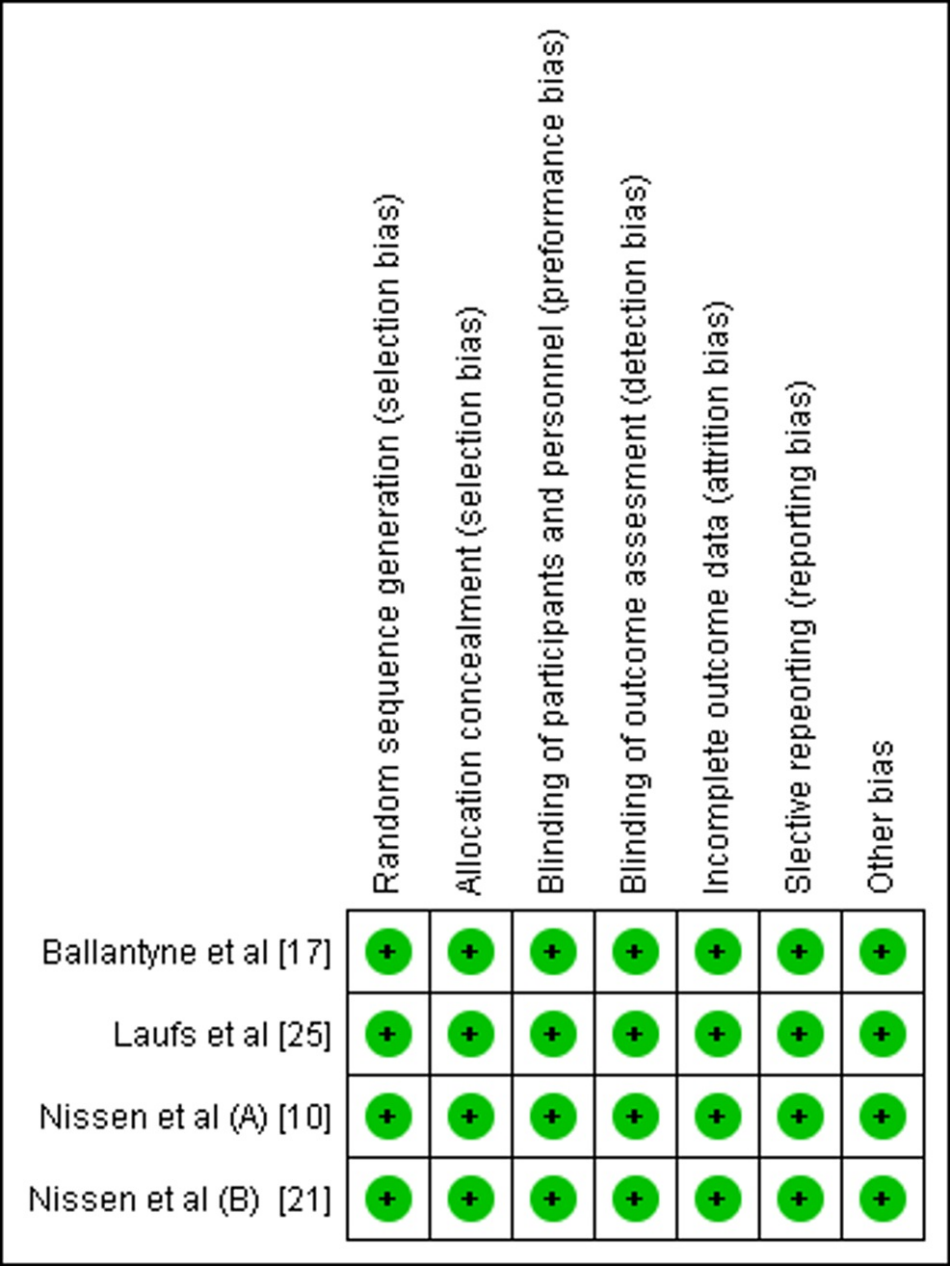
Risk-of-bias traffic light plot for studies included in the meta-analysis.

The overall risk of bias for the five studies included in the meta-analysis was low across all domains (Figures 2-3).

**Figure 4:**
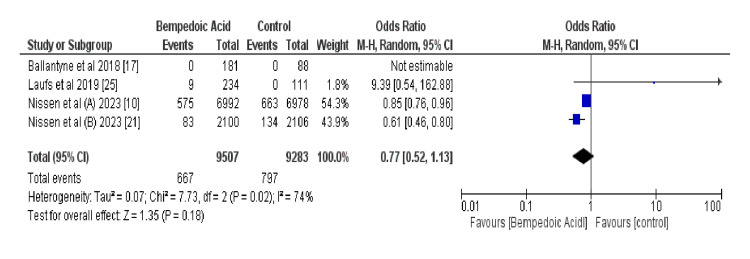
Forest plot of a three-point MACE. MACE, major adverse cardiovascular event

Figure 4 shows the pooled OR of three-point MACE outcomes of MI, stroke, and cardiovascular death using a random-effects model that incorporates all RCTs in this study. This indicates a nonsignificant trend toward benefit with bempedoic acid (odds ratio [OR] 0.77, 95% confidence interval [CI] 0.52-1.13; *P* = 0.18). Heterogeneity was moderate (*I*² = 74%), suggesting variability in the study outcomes. One study had an OR that could not be estimated, indicating potential issues with the data or rare events [10,17,21,25].

**Figure 5:**
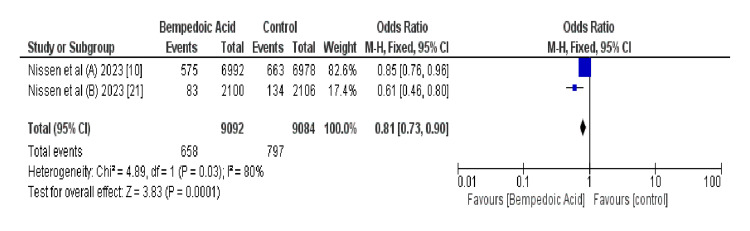
Forest plot of a four-point MACE in patients with and without previous atherosclerotic cardiovascular disease. MACE, major adverse cardiovascular event

Figure 5 shows the odds of four-point MACE, which includes MI, stroke, cardiovascular death, and unstable angina, in selected RCTs. The pooled OR of 0.81 (95% CI 0.73-0.90) suggests a statistically significant reduction in the risk of MACE with bempedoic acid. The *P*-value (<0.0001) indicates a high level of statistical significance. However, there was substantial heterogeneity (*I*² = 80%), raising concerns about the variability of outcomes across studies [10-21].

**Figure 6:**
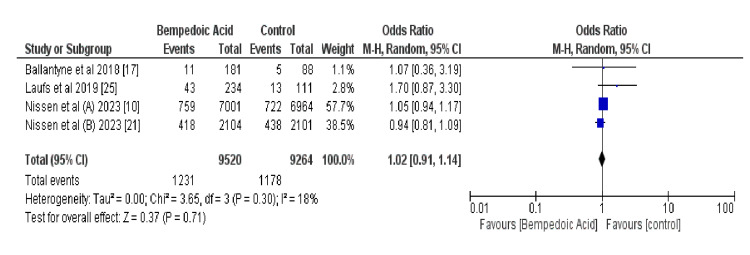
shows the data for adverse event incidence in Bempedoic acid vs. control groups

Figure 6 shows the comparative incidence of AEs between the bempedoic acid cohort and control arm across the included studies. It reports 667 events among 9,507 patients on bempedoic acid compared to 797 events in 9,283 control subjects, yielding a nonsignificant odds ratio of 0.77 (95% CI: 0.52-1.13, P = 0.18) under a random-effects model, suggestive of a non-confirmed trend towards fewer AEs with bempedoic acid [10,17, 21, 25].

Discussion

The CLEAR Serenity trial focused on evaluating the efficacy and safety of bempedoic acid in patients with statin intolerance, The trial demonstrated bempedoic acid's superiority over placebo in reducing LDL‐C in statin-intolerant patients without causing typical statin-related side effects. Notably, patients on bempedoic acid experienced fewer muscle-related symptoms compared to placebo. The lipid-lowering effect was consistent across patient groups, whether bempedoic acid was administered alone or alongside other lipid-modifying therapies. While a difference in LDL‐C reduction was observed between patients with and without DM history, it was likely due to chance, as previous trials showed comparable results regardless of diabetes status. Patients receiving bempedoic acid also exhibited significant reductions in non-HDL‐C, TC, apoB, and hs-CRP throughout the 24-week study. These reductions in atherogenic markers are crucial in reducing cardiovascular risk, as suggested by epidemiologic and genetic data. Bempedoic acid treatment was safe and well-tolerated, with no increased muscle-related AEs compared to placebo, despite acting on the same biochemical pathway as statins. The minor elevation in uric acid levels observed did not significantly lead to gout development. Adjudicated clinical events were infrequent in the bempedoic acid group, and there was no indication of heightened major adverse cardiovascular event (MACE) risk. The study's enrollment of patients typically indicated lipid-lowering therapy, had they not been intolerant to statins, reflects the high cardiovascular risk and limited treatment options for this population. It also notably included a higher percentage of women, often underrepresented in cardiovascular trials, possibly due to their increased risk for statin intolerance. However, the study's short duration limits a comprehensive assessment, and ongoing long-term studies will provide insights into extended bempedoic acid use. Overall, the CLEAR Serenity trial demonstrated the efficacy of benzoic acid in reducing LDL-C and hs-CRP in statin-intolerant patients, presenting an oral therapeutic alternative that effectively lowers LDL‐C without associated muscle-related adverse effects, complementing existing lipid-lowering therapies and providing an alternative to statins and other nonstatin therapies for managing cardiovascular risk [25].

In the CLEAR Tranquillity study involving 269 patients with statin intolerance and LDL-C levels >100 mg/dL, bempedoic acid administered at 180 mg daily for 12 weeks, alongside background ezetimibe, significantly reduced LDL-C by 28.5% and hs-CRP by 31%, demonstrating favourable outcomes for lipid and inflammatory markers. The treatment was well tolerated, with no increased discontinuation rates compared to placebo. Further trials like CLEAR Harmony, CLEAR Wisdom, and CLEAR Serenity have expanded on these findings. In CLEAR Harmony, which included 2230 high-risk patients with LDL-C >70 mg/dL despite maximally tolerated statin therapy, bempedoic acid at 180 mg daily for 52 weeks did not increase AEs or serious AEs compared to placebo. However, a higher rate of drug discontinuation and gout incidents were noted with bempedoic acid. This trial also showed a placebo-corrected LDL-C reduction of 18.1%. CLEAR Wisdom enrolled 779 high vascular-risk patients on maximally tolerated statin therapy, demonstrating that bempedoic acid at 180 mg daily for 52 weeks reduced LDL-C by 17.4% and hs-CRP by 8.7%. The CLEAR Serenity study, which included 345 patients with hypercholesterolemia and intolerance to at least two statins, reported that bempedoic acid at 180 mg daily for 24 weeks led to placebo-corrected reductions in LDL-C by 21.4% and hs-CRP by 24.3%. Importantly, there was a lower rate of myalgia compared to the placebo group. Overall, across these trials, bempedoic acid consistently demonstrated significant reductions in LDL-C and hs-CRP levels, along with favourable effects on related biomarkers, such as apoB and non-HDL cholesterol. However, while these improvements in circulating biomarkers are promising, the impact of bempedoic acid on cardiovascular outcomes is yet to be firmly established. Further research and larger trials are needed to determine the drug's effects on long-term cardiovascular events [18].

The CLEAR Serenity study revealed a notable 21.4% reduction in LDL-C levels with bempedoic acid therapy compared to placebo. In combination therapies, such as bempedoic acid plus ezetimibe, significant reductions in LDL-C have been observed. For instance, Ballantyne et al. demonstrated a substantial 38% reduction in LDL-C when bempedoic acid was added to ezetimibe in statin-intolerant subjects at high cardiovascular risk. Moreover, Rubino et al. conducted phase 2 randomized clinical trials indicating the effectiveness of triple therapy involving atorvastatin 20 mg, ezetimibe 10 mg, and bempedoic acid 180 mg [26]. This combination produced a significant reduction of 60.5% in LDL-C levels compared to placebo. Additionally, the addition of bempedoic acid to PCSK9 inhibitors (PCSK9i) exhibited a 30% reduction in LDL-C compared to placebo. These findings highlight the potential utility of bempedoic acid, especially in statin-intolerant subjects with high baseline LDL-C, such as those with familial hypercholesterolemia (FH). The ongoing CLEAR Outcomes study represents a crucial trial designed to evaluate the impact of bempedoic acid treatment on cardiovascular outcomes specifically in patients with statin intolerance. This trial aimed to provide essential insights into the cardiovascular benefits or risks associated with bempedoic acid therapy in this particular patient population [27].

A randomized controlled study based on 13,970 participants, with 6,992 receiving bempedoic acid and 6,978 receiving a placebo aimed to evaluate the impact of bempedoic acid on cardiovascular outcomes over a median follow-up period of 40.6 months [10,21]. At the beginning of the study, both the bempedoic acid group and the placebo group had a mean LDL-C level of 139.0 mg per deciliter. After six months, the reduction in LDL-C was notably greater in the bempedoic acid group compared to the placebo group, demonstrating a reduction of 29.2 mg per deciliter. This observed difference translated to a 21.1 percentage point higher reduction in LDL-C with bempedoic acid compared to the placebo. The study found significant differences in the incidence of primary endpoint events between the bempedoic acid group and the placebo group. Notably, the incidence of the primary endpoint event was lower in the bempedoic acid group (11.7%) compared to the placebo group (13.3%). Bempedoic acid also showed lower incidences in composite cardiovascular events such as death from cardiovascular causes, nonfatal stroke, or nonfatal MI, as well as fatal or nonfatal MI and coronary revascularization. However, bempedoic acid did not demonstrate significant effects on fatal or nonfatal stroke, death from cardiovascular causes, or death from any cause. AEs associated with bempedoic acid included higher incidences of gout, cholelithiasis, and small increases in serum creatinine, uric acid, and hepatic-enzyme levels compared to the placebo group. These findings suggest that while bempedoic acid showed positive outcomes in reducing certain cardiovascular events and LDL-C levels, it also presented some adverse effects that need consideration when assessing its overall benefit-risk profile for cardiovascular management [10].

This meta-analysis rigorously assessed the efficacy and safety of bempedoic acid in a statin-intolerant population, with a focus on major adverse cardiac events (MACEs) as the primary endpoint. Data were stratified into both three-point and four-point MACE outcomes to provide a thorough understanding of bempedoic acid’s cardiovascular impact. Analysis of combined study data, encompassing 667 events in 9507 subjects treated with bempedoic acid and 797 events in 9283 control subjects, revealed a nonsignificant trend favoring bempedoic acid for the reduction of three-point MACE events (OR 0.77, 95% CI 0.52-1.13, *P *= 0.18). This suggests a potential protective effect, although statistical confirmation is lacking.

A closer examination of the four-point MACE outcomes indicated a similar non-significant trend towards benefit with bempedoic acid (OR 1.02, 95% CI 0.91-1.14, *P *= 0.71), albeit with moderate heterogeneity (*I*² = 18%). Notably, the contribution from the study by Nissen et al., with a nonsignificant OR of 0.85 (95% CI 0.76-0.96) [10] was significant, whereas the results from the second study indicated a more substantial effect (OR 0.61, 95% CI 0.46-0.80) [21].

The risk-of-bias assessment, as depicted in the study graphs, indicated a low risk across most domains, reinforcing the methodological soundness of the included studies. However, this finding also highlights the need for a comprehensive evaluation of potential publication bias, which remains an underlying concern not immediately apparent from the risk-of-bias assessments.

Scientifically, the observed discrepancy between three-point and four-point MACE outcomes invites further investigation, particularly considering the complexities inherent in defining clinical endpoints. The inclusion of subjective components such as unstable angina in the four-point MACE definition could contribute to heterogeneity, illustrating the challenges in classifying clinical events within the context of a trial. Clinically, the results suggest that while bempedoic acid may demonstrate a tendency towards cardiovascular benefit, the evidence does not conclusively support its efficacy in reducing MACE outcomes. This necessitates a cautious approach to its application, advocating for a personalized assessment of its role in the management of hyperlipidemia in patients with statin intolerance. Future research, informed by subgroup analyses, could be instrumental in elucidating the impact of bempedoic acid, particularly when considering the varying baseline cardiovascular risks and doses or comparators used in control arms. Such detailed analyses could offer a more nuanced understanding of the therapeutic effects.

A recent update was that the US Food and Drug Administration (FDA) has expanded the indications for bempedoic acid (Nexletol) and bempedoic acid plus ezetimibe combination (Nexlizet) to prevent heart attacks and cardiovascular procedures in both primary- and secondary-prevention patients regardless of statin use. According to manufacturer Esperion, bempedoic acid is now indicated to reduce the risk for MI and coronary revascularization in adults who are unable to take recommended statin therapy (including those not taking a statin) with established CVD or who are at high risk for a CVD event but without established CVD [27,28].

In summary, although the current meta-analysis does not demonstrate a significant impact of bempedoic acid on MACE reduction in statin-intolerant individuals, the low to moderate heterogeneity and minimal risk of bias observed in the studies offer a strong basis for future research. Subsequent studies should focus on clarifying the effects of dosage, comparators, and patient risk profiles on the efficacy of bempedoic acid, potentially leveraging individual patient data to provide more detailed insights. Two studies from these four were part of the same larger trial known as the CLEAR Outcomes trial. Both articles discussed the effects of bempedoic acid on cardiovascular outcomes in statin-intolerant patients, but they targeted slightly different aspects and presentations of the data. The *New England Journal of Medicine* (NEJM) study is more general and discusses the overall trial results, including a broader spectrum of participants (both primary and secondary prevention groups) [10]. The *Journal of American Medical Association* (JAMA) article focuses specifically on the primary prevention subgroup within the same trial [21], which summarizes the key differences and similarities, highlighting that, while the core dataset and trial design might be similar, the presentations and focus of the analyses differ, catering to different clinical questions and audience insights. Both articles were derived from the same larger dataset, emphasizing different aspects of the trial's outcomes based on the target population and specific endpoints [10,21].

## Conclusions

Bempedoic acid operates as an inhibitor of adenosine triphosphate-citrate lyase (ACLY), a crucial enzyme in cholesterol synthesis. This drug is a pro-drug that undergoes conversion into its active form, bempedoic acid CoA, facilitated by very long-chain acyl-CoA synthetase 1 (ASCVL1), predominantly localized in the liver, developing muscle-related side effects commonly associated with other cholesterol-lowering medications. In concluding this meta-analysis, it's clear that while bempedoic acid does not significantly reduce MACEs for statin-intolerant patients, there remains a hint of potential cardiovascular benefit.

Bempedoic acid ezetimibe, as an adjunct to diet, is indicated alone or in combination with other LDL-C-lowering therapies for reducing LDL-C levels in adults with primary hyperlipidemia, including heterozygous familial hypercholesterolemia (HeFH). It is also indicated in cases where concomitant LDL-C-lowering therapy is not feasible for reducing LDL-C levels in adults with primary hyperlipidemia, including HeFH. Bempedoic acid's role in dyslipidemia management is supported by its efficacy in achieving significant lipid reductions, thereby potentially lowering the risk of cardiovascular events in statin-intolerant patients. Therefore, it serves as an addition to the currently available lipid-lowering therapy, enabling more personalized and effective treatment strategies for individuals with dyslipidemia. Further research and RCTs will continue to refine its application, ensuring optimal outcomes for patients.
